# Fabrication of WO_3_·2H_2_O/BC Hybrids by the Radiation Method for Enhanced Performance Supercapacitors

**DOI:** 10.3389/fchem.2018.00290

**Published:** 2018-08-13

**Authors:** Fan Yang, Jinzhi Jia, Rui Mi, Xichuan Liu, Zhibing Fu, Chaoyang Wang, Xudong Liu, Yongjian Tang

**Affiliations:** ^1^Science and Technology on Plasma Physics Laboratory, Research Centre of Laser Fusion, China Academy of Engineering Physics, Mianyang, China; ^2^School of Materials Science and Engineering, Southwest University of Science and Technology, Mianyang, China; ^3^College of Materials Science and Engineering, Chongqing University, Chongqing, China

**Keywords:** γ-irradiation method, WO_3_·2H_2_O/BC hybrids, higher specific capacitance, cyclic stability, supercapacitors

## Abstract

In this study, we described a facile process for the fabrication of tungsten oxide dihydrate/bamboo charcoal hybrids (WO_3_·2H_2_O/BC) by the γ-irradiation method. The structural, morphological, and electrochemical properties of WO_3_·2H_2_O/BC hybrids were investigated using X-ray diffraction (XRD), field emission scanning electron microscopy (FESEM), transmission electron microscopy (TEM), cyclic voltammetry (CV), galvanostatic charge/discharge (GCD), and electrochemical impedance spectroscopy (EIS) techniques. The combination of BC (electrical double layer charge) and WO_3_·2H_2_O (pseudocapacitance) created a combined effect, which enhanced the specific capacitance and superior cyclic stability of the WO_3_·2H_2_O/BC hybrid electrode. The WO_3_·2H_2_O/BC hybrids showed the higher specific capacitance (391 F g^−1^ at 0.5 A g^−1^ over the voltage range from −1 to 0 V), compared with BC (108 F g^−1^) in 6 M KOH solution. Furthermore, the hybrid electrode showed superior long-term performance with 82% capacitance retention even after 10,000 cycles. The experimental results demonstrated that the high performance of WO_3_·2H_2_O/BC hybrids could be a potential electrode material for supercapacitors.

## Introduction

For the rapid increase of global energy demand and the depletion risk of fossil fuels, developing alternative sustainable, affordable, efficient, and clean energy has become very important (Zhao Y. et al., 2016, 2017; Xu et al., 2017; Yi et al., 2017; Zhao B. et al., 2017). Among energy storage devices, supercapacitors (or ultracapacitors) are promising, owing to their safe operation, super-high service life, and great power density (Wang K. et al., 2014; Dai et al., 2017; Zhao B. et al., 2017; Zhao et al., 2018). They have wide application areas such as electric vehicles, pulse power systems and portable devices (Wang et al., 2018). Depending on the charge storage mechanism, supercapacitors are generally classified into electrical double layer charge (EDLC) and pesudoprocess charge storage. The former stores charges electrostatically in double layers, whereas the latter stores charges on the surface of the electrode active materials as faradaic redox reactions (Zhang and Park, 2017). In general, carbon-based materials are EDLC type (Pang et al., 2016), whereas transition metal oxides and conducting polymers are pseudocapacitor-type materials (Zhang et al., 2012; Yao C. et al., 2017; Yao S. et al., 2017).

The charge storage of pesodocapacitors is much higher compared to those of EDLCs. More recently, transition metal oxides (i.e., RuO_2_, V_2_O_5_, NiO, MnO_2_, SnO_2_, and WO_3_) have been widely investigated in the applications for supercapacitors, and their charge storage originates from fast superficial redox reactions (Zhu and He, 2012; Zhang et al., 2015; Qiu et al., 2016; Zeng et al., 2017; Zhang and Park, 2017; Liu et al., 2018a). Among many of the reported transition metal oxides, RuO_2_ has been considered as the proper material with excellent capacitive performance. However, RuO_2_ is expensive and rare, constraining the wide practical applications in electrode materials (Cai et al., 2014). WO_3_ is a promising electrode material owing to its various morphologies, high theoretical specific capacitance, environmental friendliness, and low cost (Qiu et al., 2016). Nevertheless, its low electrical conductivity (10^−5^–10^−6^ S cm^−1^) has limited the wide applications. If we can improve the conductivity of WO_3_, higher specific capacitances could be achieved as expected. Therefore, many researchers have focused on incorporating WO_3_ with highly conductive carbon materials to establish a hybrid-type material that combines advantages of each component (Lu et al., 2012; Xiao et al., 2012; Reddy et al., 2015; Sun et al., 2015; Wang et al., 2015; Yuksel et al., 2016). Wang et al. fabricated the WO_3_/carbon aerogel hybrids with outstanding long-term stability, and the specific capacitance was ~700 F g^−1^ (at a scan rate of 25 mV s^−1^ in 0.5 M H_2_SO_4_ over a voltage window of −0.3 to 0.5 V; Wang Y. H. et al., 2014). Huang et al. also designed graphene-WO_3_ hybrids with enhanced supercapacitor capacitance (Xing et al., 2016). Wang et al. fabricated the graphene nanosheets-tungsten oxides with high supercapacitor performance (Wang et al., 2015). Ma et al. fabricated a hybrid based on graphene and WO_3_ via the hydrothermal method, which possessed high specific capacitance and superior rate capability (Ma et al., 2015).

Among the conductive materials, activated carbon-based materials are the most promising candidates for supercapacitor applications, due to their unique characteristics of large surface areas, and high electrochemical stability and conductivity (Yang et al., 2014; Li and Wu, 2015; Wang et al., 2016; Boyjoo et al., 2017; Dai et al., 2018). In carbon materials, bamboo charcoal draws research attention for its extraordinarily porous microstructure, cost efficiency, and high absorptive capacity (Wang et al., 2012, 2013, 2016; Zhang et al., 2013; Yang et al., 2014; Li and Wu, 2015; Yu et al., 2015). Li et al. studied the water bamboo-derived porous carbon with a maximum specific capacitance of 268 F g^−1^ at a current density of 1 A g^−1^ in 6 M KOH electrolyte and good capacity retention of 97.28% even over 5,000 cycles at a current density of 10 A g^−1^ (Li and Wu, 2015). Yang et al. also synthesized BC by KOH activation, and the specific capacity retention was more than 91% after 3,000 cycles (Yang et al., 2014). Impressively, BC has long-lasting life, whereas WO_3_ has high theoretical specific capacity. For this respect, combining each advantage of BC and WO_3_·2H_2_O to improve the performance might bring novel and excellent properties. However, so far, to the best of our knowledge, nearly no works have been done in this aspect.

For decades, researchers have reported many ways to fabricate WO_3_/carbon materials, such as hydrothermal method, impregnation methods, and sol-gel method. Compared with these methods, as we previously reported, the radiation method can improve the contact between the doped materials and pristine carbon, and this method has been successfully applied in H_2_ storage (Zhong et al., 2015, 2016). Obviously, good adhesion may improve the stability of hybrids and optimize the conduction of electrons, which will enhance the capacitive properties of the hybrids. But so far, no studies have been conducted on the preparation of metal oxides and carbon hybrid materials for supercapacitor applications by the irradiation method. To widen application of this method and further improve the capacitive performance, it is important to develop this method.

In this work, novel WO_3_·2H_2_O/BC hybrids were designed and fabricated by a facile γ-irradiation strategy. Morphologies and microstructures of the samples were investigated by XRD, SEM, TEM, and XPS, whereas CV, GCD, and EIS were carried out to study capacitive properties. The electrochemical results demonstrated that WO_3_·2H_2_O/BC hybrids delivered a high specific capacity (391 F g^−1^ at 0.5 A g^−1^) and superior long-term stability (82% retention even after 10,000 cycles). The combination of bamboo (EDLC) and WO_3_·2H_2_O (pseudocapacitance) provided short ion diffusion path and fast electron transport, leading to a great supercapacitor.

## Experimental details

### Synthesis of WO_3_·2H_2_O/BC hybrids

**Figure 1:**
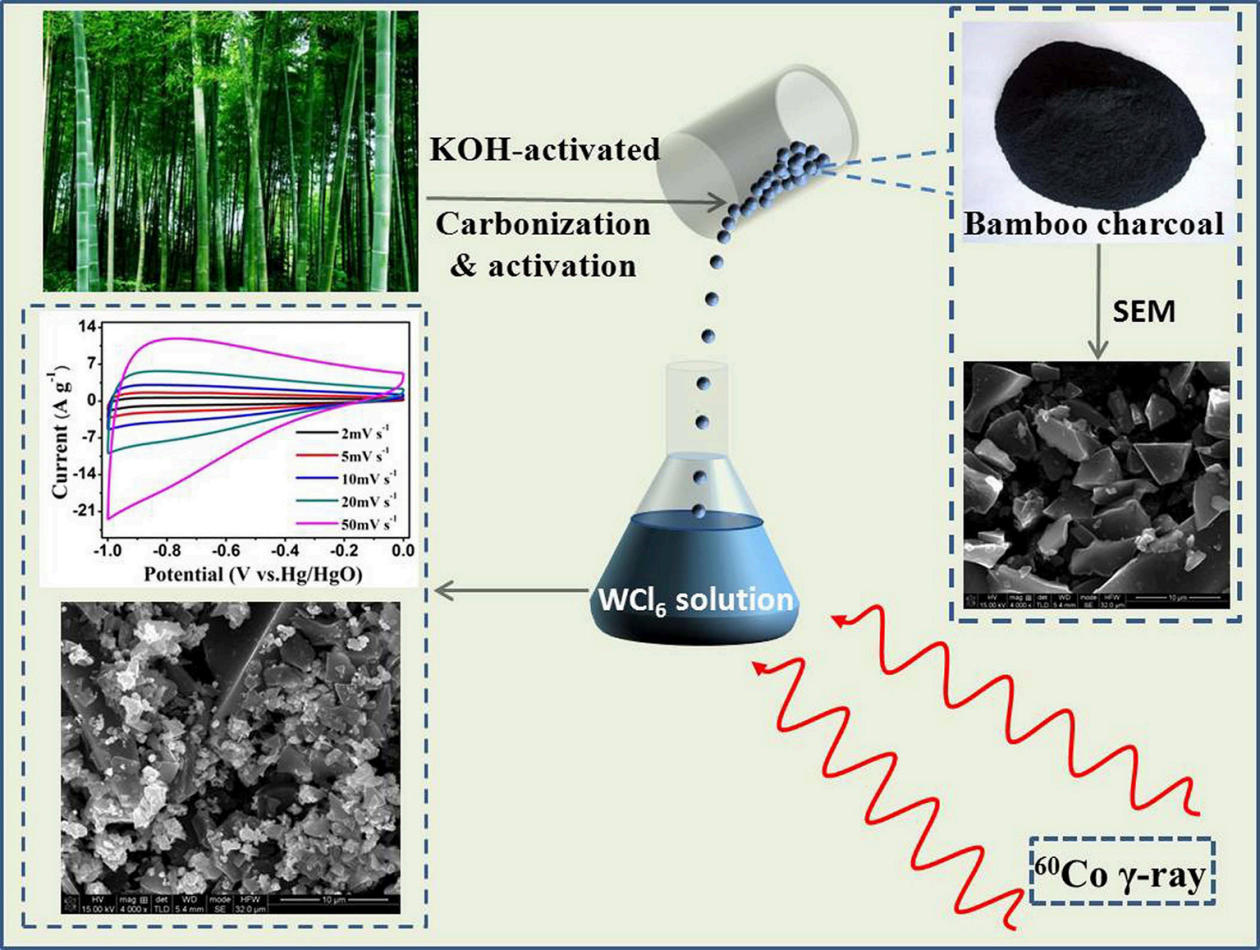
Schematic diagram for the synthesis of WO_3_·2H_2_O/BC hybrids and their supercapacitor performance.

All the reagents and solvents were analytical grade and used without further purification. In a typical process, WO_3_·2H_2_O/BC hybrids were prepared as follows. WCl_6_ (3 mg) was added into isopropyl alcohol (20 mL) under stirring for 20 min in a glass vial at room temperature. Then, the BC monoliths (0.2 g) were slowly impregnated with 10 ml of WCl_6_ solution. After 30 min of continuous stirring, 2-propanol was added with proper amount to scavenge H^*^ and OH^*^ radicals, which were generated during irradiation. The mixture was irradiated at room temperature with a ^60^Co γ-ray source at a dose rate of 200 Gy min^−1^, and the total dose was 500 kGy. The product was collected by centrifugation and rinsed several times with DI water and ethanol, and then dried at 60°C for 12 h. Figure 1 shows the schematic diagram for the synthesis of WO_3_·2H_2_O/BC hybrids and their supercapacitor performance. For comparison, bamboo charcoal was prepared by carbonization of natural bamboo with the KOH-modified method, as described elsewhere (Yang et al., 2014; Li and Wu, 2015).

### Material characterization

The crystalline phase of WO_3_·2H_2_O/BC hybrids was examined by X-ray powder diffraction (XRD) employing monochromatized CuKα incident radiation. The morphology and microstructure were analyzed by using a field emission scanning electron microscope (FESEM, Nova 600i) with an attached energy dispersive X-ray spectroscopy (EDS) analysis and transmission electron microscopy (TEM). X-ray photoelectron spectroscopy (XPS) was studied for detecting chemical composition and oxidation states of WO_3_·2H_2_O/BC hybrids.

### Electrochemical measurements

Electrochemical tests were carried out at room temperature in a conventional three-electrode configuration with 6 M KOH as an electrolyte using an electrochemical workstation (CHI 660E), WO_3_·2H_2_O/BC as a working electrode, platinum foil as a counter electrode, and Hg/HgO as a reference electrode. In electrochemical tests, WO_3_·2H_2_O/BC (80%) was mixed with acetylene black (10%) and polyvinylidene fluoride (PVDF, 10%) in N-methyl-2-pyrrolidone (NMP) to form slurry. Then the slurry was coated on glassy carbon to fabricate the working electrode. The potential range for CV tests is from −1 to 0 V, and the measurement range for EIS tests is between 0.1 Hz and 100 kHz with an AC amplitude of 5 mV.

## Results and discussion

### Characterization of WO_3_·2H_2_O/BC hybrids

**Figure 2:**
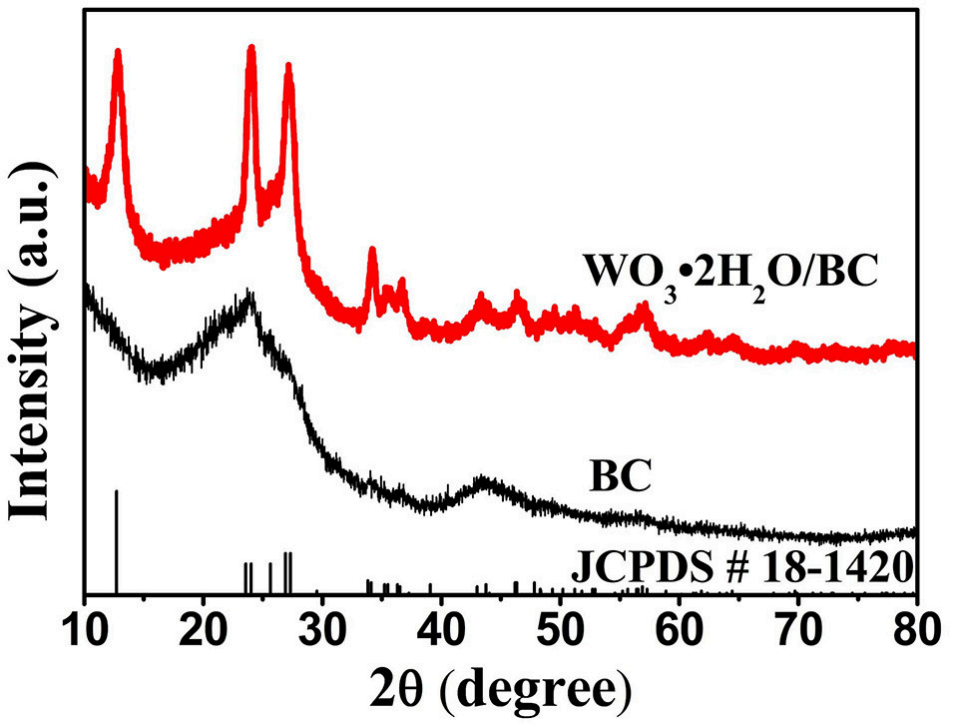
XRD patterns of the BC and WO_3_·2H_2_O/BC hybrids.

Crystal structures of the as-prepared BC and WO_3_·2H_2_O/BC hybrids were first studied by XRD. As shown in Figure 2, two broad peaks near 23 and 43° correspond to (002) and (100), respectively, which can be identified for the amorphous forms of BC. For WO_3_·2H_2_O/BC hybrids, two broad peaks of BC also appeared, and all the peaks exclusively assigned to the characteristic structure of WO_3_·2H_2_O (JCPDS-00-18-1420). Furthermore, no other impurity phase peak can be detected, and the existence of strong and sharp peaks also indicates that WO_3_·2H_2_O/BC hybrids have high crystallinity. The structure of WO_3_·2H_2_O has been reported to be attractive as electrode materials (Ma et al., 2015; Li et al., 2016; Mitchell et al., 2017). Crystalline WO_3_ is much more stable than amorphous WO_3_ due to the denser structure and lower dissolution rate in electrolytes, which is a very important point in terms of practical applications (Liu et al., 2017). The diffraction pattern of WO_3_·2H_2_O/BC hybrids is the combination of the peaks from BC and WO_3_·2H_2_O, demonstrating the successful composite. However, for the amorphous forms of BC, no distinct peaks of BC can be observed in WO_3_·2H_2_O/BC hybrids. Nevertheless, the presence of BC in the WO_3_·2H_2_O/BC hybrids can be confirmed by the results of SEM, TEM, and XPS.

**Figure 3:**
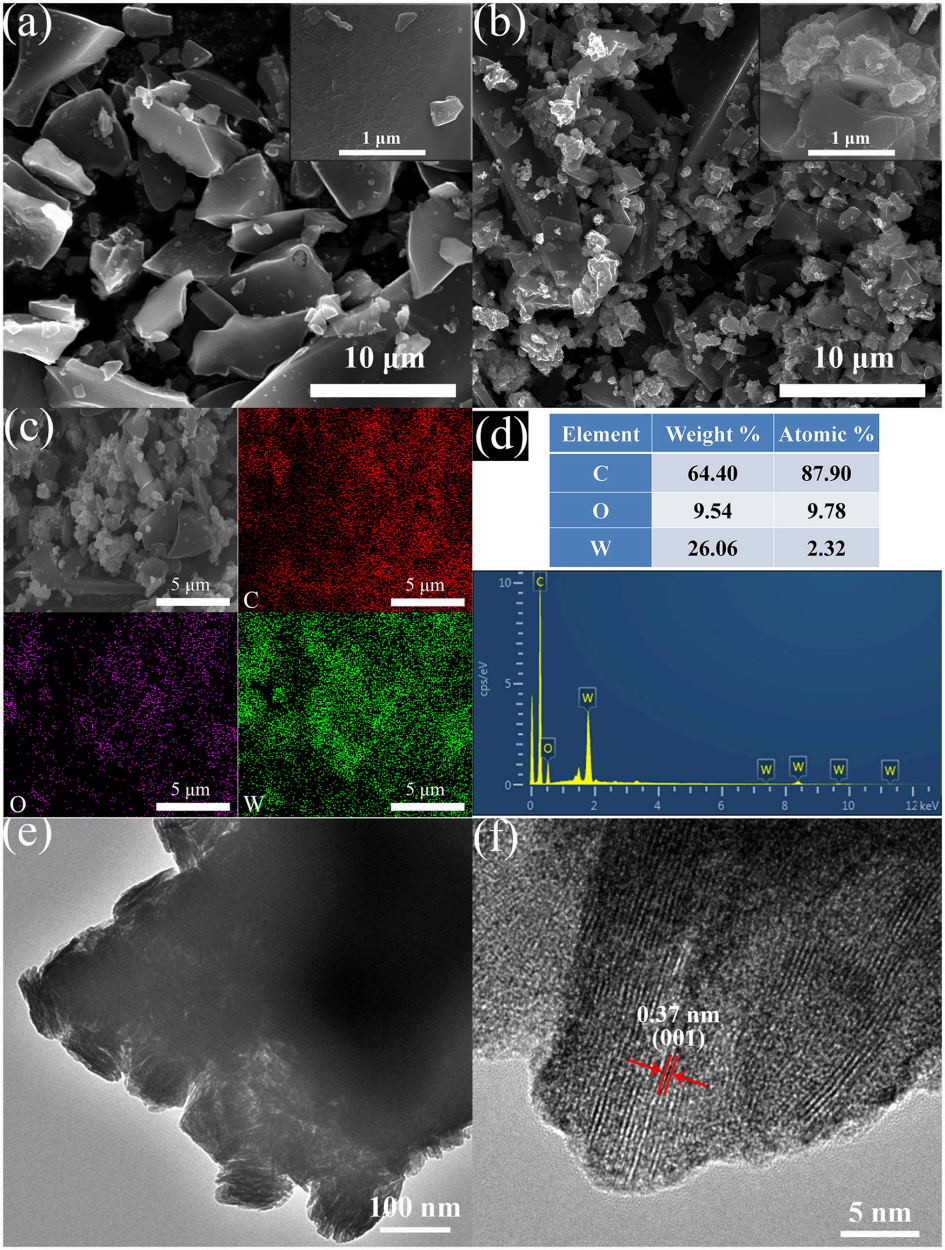
Typical SEM images of **(a)** BC and **(b)** WO_3_·2H_2_O/BC hybrids. **(c)** The EDS mapping results and **(d)** the corresponding spectrum for the WO_3_·2H_2_O/BC hybrids. **(e)** TEM and **(f)** HRTEM images of the WO_3_·2H_2_O/BC hybrids.

The surface morphologies of BC and WO_3_·2H_2_O/BC hybrids were characterized by SEM and TEM. As shown in Figure 3a, we can clearly see that the BC has smooth surface and irregular forms. Figure 3b shows the morphology of WO_3_·2H_2_O/BC hybrids, and the skeleton of BC can be clearly seen with a random distribution of WO_3_·2H_2_O. EDS mapping was further used to demonstrate the formation of WO_3_·2H_2_O/BC hybrids, and is shown in Figure 3c. Obviously, the C, W, and O elements exist in WO_3_·2H_2_O/BC hybrids, and this result is in accord with the EDS spectrum shown in Figure 3d. As shown in Figures 3e,f, TEM and high-resolution TEM (HRTEM) micrographs further indicate microscopic structures of WO_3_·2H_2_O/BC hybrids. The TEM image of WO_3_·2H_2_O/BC hybrids clearly reveals that WO_3_·2H_2_O is successfully connected to BC. The HRTEM image of WO_3_·2H_2_O/BC (Figure 3f) shows that the spacing between adjacent lattice planes is 0.37 nm, corresponding to the (001) plane of WO_3_·2H_2_O, indicating that WO_3_·2H_2_O grows preferentially along (001) (Mitchell et al., 2017). Therefore, WO_3_·2H_2_O/BC hybrids can be successfully synthesized via a simple γ-irradiation method, in which WO_3_·2H_2_O is sufficiently connected to BC. And the robust contact between WO_3_ and BC can be maintained by ultrasound for 30 min without WO_3_ shed (see Figure 3e). This great interfacial contact between WO_3_·2H_2_O and BC may be possibly favorable for the electronic transport process, thus resulting in the enhanced supercapacitor performance (Cai et al., 2014; Chu et al., 2017; Liu et al., 2018a,b). To further check the surface chemical composition of WO_3_·2H_2_O/BC hybrids, XPS measurements were carried out.

**Figure 4:**
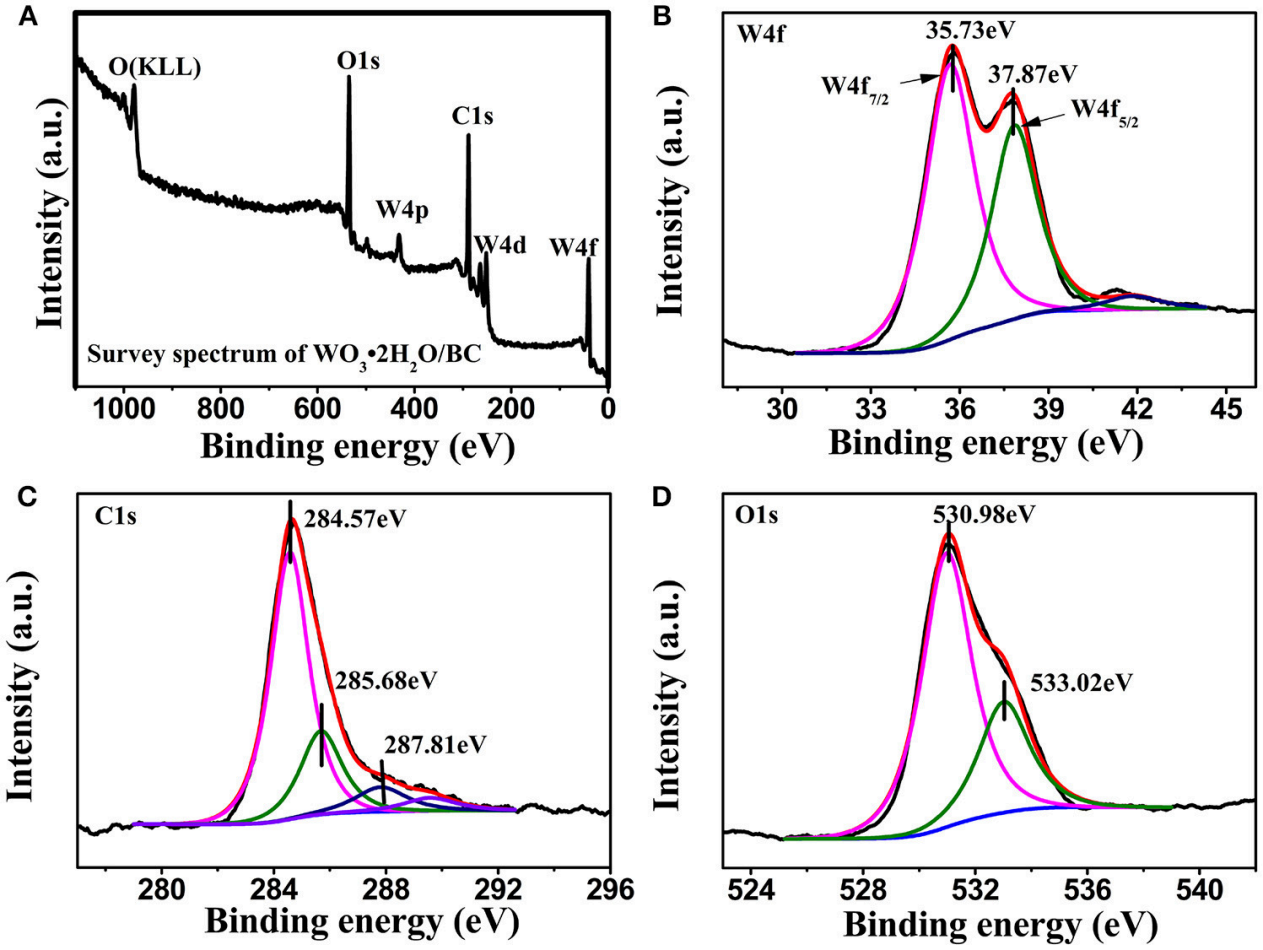
The XPS spectra of WO_3_·2H_2_O/BC **(A)** survey scan spectrum, **(B)** W 4f core level spectrum, **(C)** C 1s core level spectrum, and **(D)** O 1s core level spectrum.

The detailed composition and surface valence state of WO_3_·2H_2_O/BC hybrids was probed by XPS measurements. Figure 4A shows the survey scan spectrum of the WO_3_·2H_2_O/BC hybrids, and only W, O, and C elements exist in the hybrids without evidence of any other impurity atoms. Figure 4B shows the XPS spectrum of the W 4f doublet peak in the high-resolution scan. The peaks located at 37.87 and 35.73 eV are attributable to the W 4f_5/2_ and W 4f_7/2_, respectively. The observed energy position of the doublet is in accord with the previous report for the W^6+^ oxidation state (Shinde et al., 2016; Xu et al., 2016; Liu et al., 2017; Wu and Yao, 2017). The splitting between W 4f_7/2_ and W 4f_5/2_ is 2.14 eV, demonstrating a typical state of W^6+^ in WO_3_·2H_2_O/BC hybrids, which is well analogous to the XRD study. The XPS spectrum of C 1s from the WO_3_·2H_2_O/BC hybrids (see Figure 4C) is also decomposed into three peaks at 284.57 eV (C–C), 285.68 eV (C–O), and 287.81 eV (C = O), suggesting that the bonding between carbon atoms of BC and oxygen atoms of WO_3_·2H_2_O improves the conductivity and accelerates charge transport for the hybrids (Cai et al., 2014; Nayak et al., 2017). And the deconvolution peak of the O 1s spectrum can be resolved into two components of 530.98 and 533.02 eV (see Figure 4D). The low binding energy component at 530.98 eV is attributed to the O^2−^ bond with wolfram, and the latter peak is assigned to OH^−^ (Xing et al., 2016; Nayak et al., 2017).

### Electrochemical performance of WO_3_·2H_2_O/BC hybrids

**Figure 5:**
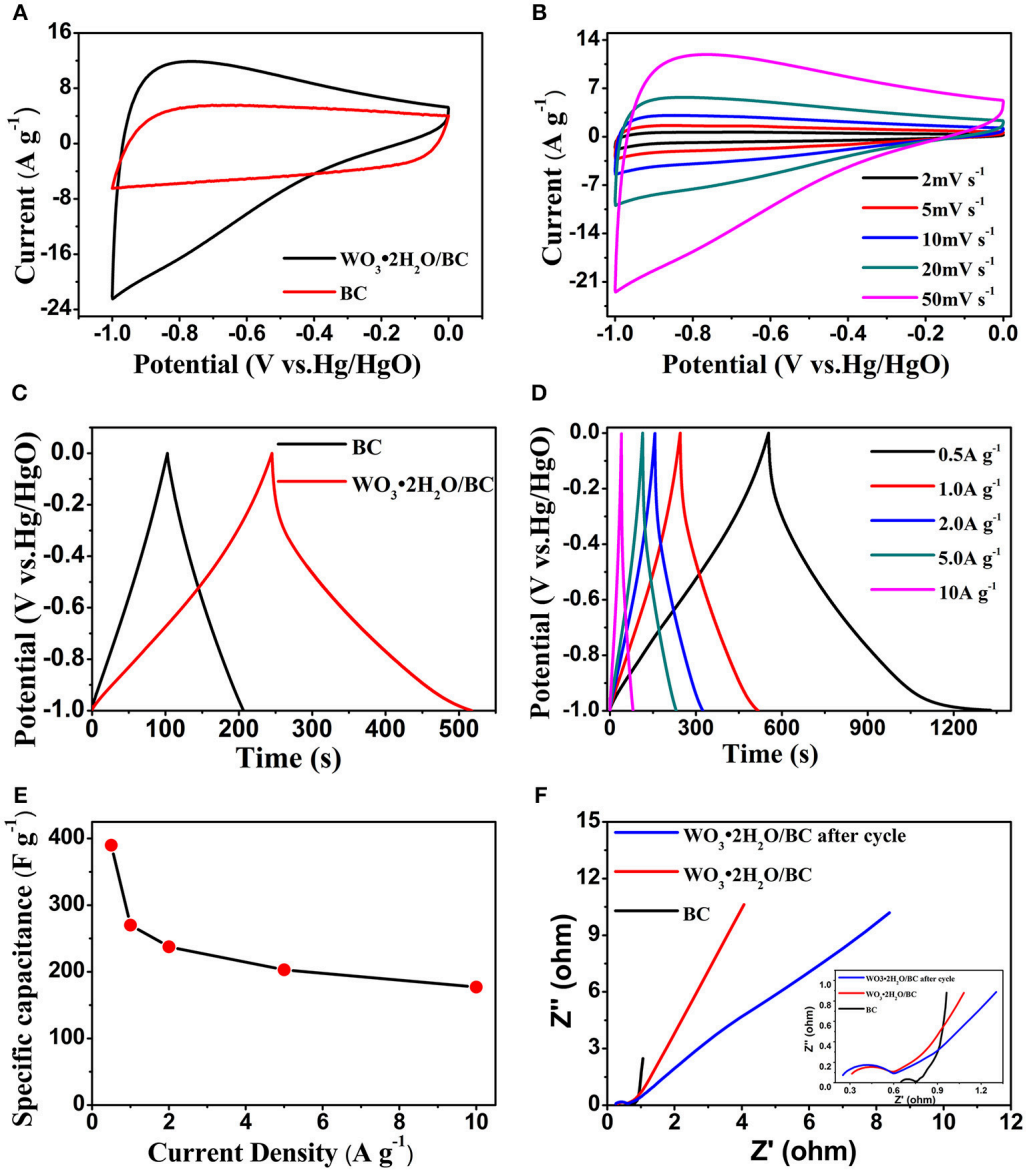
**(A)** The CV curves of BC and WO_3_·2H_2_O/BC electrodes at a scan rate of 50 mV s^−1^. **(B)** The CV curves of the WO_3_·2H_2_O/BC electrode at different scan rates. **(C)** GCD curves of BC and WO_3_·2H_2_O/BC electrodes at a current density of 1 A g^−1^. **(D)** GCD curves of the WO_3_·2H_2_O/BC electrode at different current densities. **(E)** Specific capacitance of the WO_3_·2H_2_O/BC electrode at different current densities. **(F)** EIS of the BC and WO_3_·2H_2_O/BC electrodes.

The electrochemical performance of WO_3_·2H_2_O/BC hybrids was first investigated by the CV test. The CV curves of the BC and WO_3_·2H_2_O/BC electrodes are shown in Figure 5A. Compared with those of BC, the CV curves of WO_3_·2H_2_O/BC changed from the rectangular shape to the “dolphin-like” shape, and nearly no obvious redox peaks detected are characteristic among various WO_3_ (Reddy et al., 2015; Thind et al., 2016; He et al., 2017; Nayak et al., 2017). Moreover, the stored charge of the hybrids can be calculated by the enclosed area of the CV curve. The observed integrated area of the WO_3_·2H_2_O/BC electrode at the same current density is much larger than that of the BC electrode, suggesting the contribution of WO_3_·2H_2_O incorporation to the enhanced specific capacitance of WO_3_·2H_2_O/BC, and the combined effect of WO_3_·2H_2_O and BC is significant (Cai et al., 2014; Liu et al., 2018a). The results obtained here are also consistent with the SEM and TEM morphologies, suggesting that the WO_3_·2H_2_O/BC hybrid morphology provides good contact fascinating the fast charge intercalation/deintercalation process, and the enhanced electronic conductivity after carbon incorporation contributes to the superior electrochemical performance. Figure 5B shows the CV curves of WO_3_·2H_2_O/BC in 6 M KOH at different scan rates (2, 5, 10, 20, and 50 mV s^−1^) over a potential window of −1 to 0 V. The corresponding CV curves of BC are provided in Figure S1a. The increased area under the curve with scan rate is clearly observed, indicating an excellent capacitance behavior and high-rate capability of the electrode.

Charge-discharge measurements were conducted under galvanostatic conditions at different applied current densities. The GCD plots of the BC and WO_3_·2H_2_O/BC electrodes at a current density of 1 A g^−1^ are presented in Figure 5C. According to the galvanostatic discharge curves, the specific capacitance (*C*_s_, F g^−1^) of the electrode is calculated according to the following equation:

$$\begin{array}{l} C_{s}\left(Fg^{-1}\right)=\frac{I\Delta t}{m\Delta V} \end{array}$$

where *I* (mA) represents the applied current, Δ*t* (s) the discharge time, Δ*V* (V) the potential window, and *m* (mg) the weight of the active material. In addition, the discharge time of WO_3_·2H_2_O/BC is much larger than that of BC, showing higher capacitance. And this result is also consistent with the CV tests. GCD curves of WO_3_·2H_2_O/BC and BC (Figure S1b) electrodes recorded at 0.5, 1, 2, 5, and 10 A g^−1^ are shown in Figure 5D. With the increasing charging and discharging currents, a highly linear and nearly symmetric relationship between the potential and time was also observed, suggesting the desired fast charging and discharging property of the materials. No obvious internal resistance (IR) drop of the BC (Figure S1b) and WO_3_·2H_2_O/BC electrodes was observed for any of the curves, which indicates high conductivity of the materials. The higher *C*_s_ of WO_3_·2H_2_O/BC is due to the combined effects between WO_3_·2H_2_O and BC (Chu et al., 2017), and the superior electrochemical performance may be mainly attributed to the enhanced electronic conductivity after carbon incorporation. As shown in Figure 5E, the calculated *C*_s_ of WO_3_·2H_2_O/BC are 391, 270, 227.5, 203, and 177 F g^−1^ at current densities of 0.5, 1, 2, 5, and 10 A g^−1^, respectively, demonstrating that the *C*_s_ decreases with increasing current density (Shinde et al., 2017). The coulombic efficiency of WO_3_.2H_2_O/BC hybrids is ~100%, exhibiting a good reversibility during the charge/discharge process. Furthermore, ~45% of the capacitance was retained when the current density increased from 0.5 to 10 A g^−1^, higher than the reported graphene nanosheets-tungsten oxides composites (34% capacitance retention from 0.1 to 5 A g^−1^), owing to the sluggish intercalation/deintercalation process of WO_3_.2H_2_O/BC at a high scan rate (Cai et al., 2014). These results are comparable to those of WO_3_-carbon-based electrodes in earlier reports (Table S1). To sum up, the enhancement in the electrochemical performance for WO_3_·2H_2_O/BC hybrids is mainly explained as follows: (i) the great interfacial contact between WO_3_·2H_2_O and BC provides short ion diffusion paths and the rapid electronic transports; (ii) the superior electrochemical performance may be mainly attributed to the enhanced electronic conductivity after carbon incorporation.

The EIS study was conducted to elucidate electrical conductivity and ion transfer features of BC and WO_3_·2H_2_O/BC electrodes. Electrochemical impedance characteristics of the electrode were investigated in a frequency range from 100 kHz to 0.1 Hz with an AC amplitude of 5 mV in 6 M KOH electrolyte. Figure 5F displays the typical Nyquist plots of these BC and WO_3_·2H_2_O/BC samples and the inset figure shows the magnified plots. For all the Nyquist plots, a semicircle can be seen in the high-frequency region, whereas in the low-frequency region, a straight line can be found. According to the previous reports (Sun et al., 2015; Chu et al., 2017; Yao C. et al., 2017), a straight line should relate to the ion diffusion into the active electrode (Zw), whereas the semicircle can be assigned to the charge transfer resistance (Rct) owing to the faradic and non-faradic reactions at the electrode/electrolyte interface. After doped with WO_3_·2H_2_O, the WO_3_·2H_2_O/BC electrode exhibits larger Rct and Zw than BC, mainly due to the poor electric conductivity of WO_3_.2H_2_O (Shinde et al., 2017). For comparison of first cycle and after 10,000 cycles, the Rct of WO_3_·2H_2_O/BC hybrids after the cycle is slightly larger than the value before the cycle, suggesting that WO_3_·2H_2_O/BC hybrids have good stability (Cai et al., 2014).

**Figure 6:**
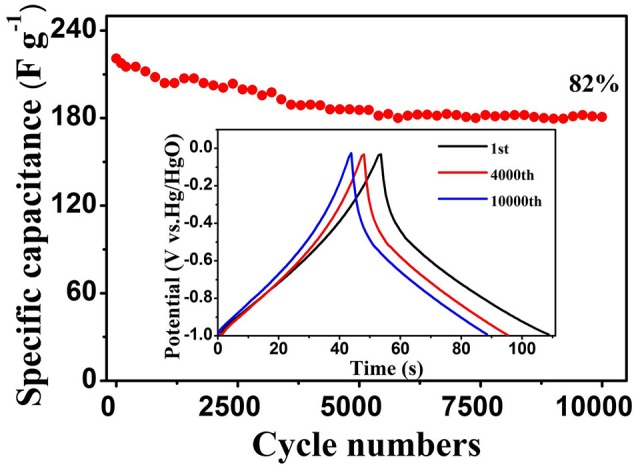
Cycling stability of the WO_3_·2H_2_O/BC electrode at 4 A g^−1^; the inset shows GCD curves at different cycles.

The cycling stability is the key factor to evaluate the practical applications of electrodes. To explore this, the cycle stability of WO_3_·2H_2_O/BC hybrids was further investigated by repeating the GCD test between −1 and 0 V at 4 A g^−1^ for 10,000 cycles (Figure 6). Impressively, after 10,000 cycles, the initial Cs (220.8 F g^−1^) of the WO_3_·2H_2_O/BC electrode slightly declined to 180.8 F g^−1^, and approximately 82% of the initial capacitance was retained. The inset shows the 1st, 4,000th and 10,000th GCD curves, respectively, indicating that the GCD profiles retain superior linearity and symmetry even after 10,000 cycles. Compared with other studies, to the best of our knowledge, in our work, the WO_3_·2H_2_O/BC hybrid electrode shows comparable cycling stability (Table S1); moreover the BC used in our work is much cheaper. Therefore, the combined effect of WO_3_·2H_2_O and BC accelerates the sufficient ion diffusion. This result confirms that the prepared WO_3_·2H_2_O/BC hybrids were highly stable as a novel supercapacitor electrode.

**Figure 7:**
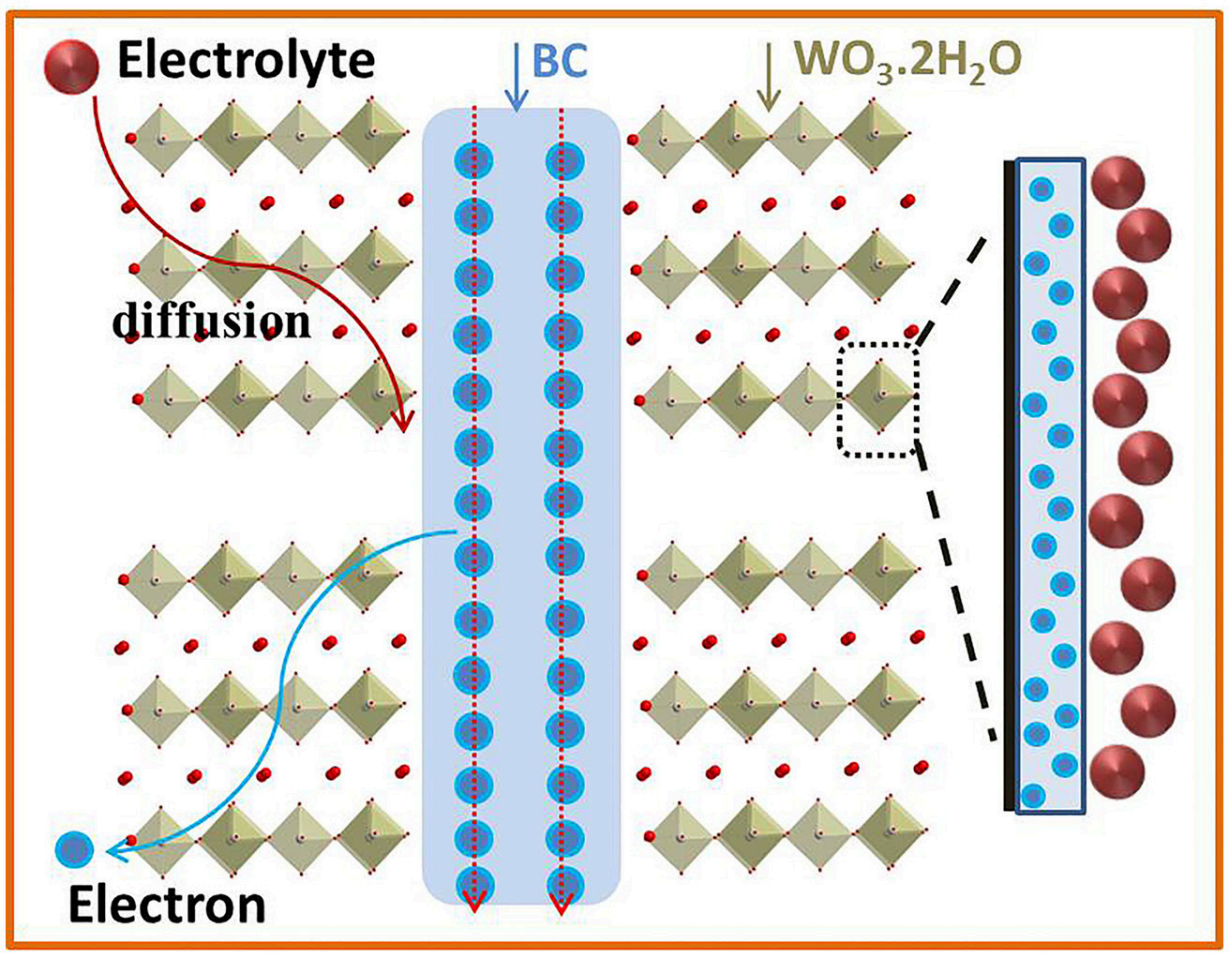
Anchored WO_3_.2H_2_O crystals with conductive BC; both electron and electrolyte can access WO_3_.2H_2_O surfaces, and an enhanced pseudocapacitive process can be formed on the surface of WO_3_.2H_2_O/BC.

Figure 7 shows the storage mechanism of WO_3_·2H_2_O/BC hybrids. Briefly, the mechanism of WO_3_·2H_2_O/BC hybrids can be described as follows. First, the high conductivity of BC facilitates the rapid transfer of electrons. Secondly, after the γ-irradiation process, the good contact of BC and WO_3_·2H_2_O guarantees a low internal resistance between BC and WO_3_·2H_2_O, and also facilitates the transmission of low-loss electrons to the anchored WO_3_·2H_2_O. Thirdly, the structure of WO_3_·2H_2_O facilitates ion adsorption and insertion in the electrolyte, so an enhanced pseudocapacitor process can be formed on the surface of WO_3_·2H_2_O. In addition, the good contact of BC and WO_3_ also makes it difficult for WO_3_·2H_2_O to agglomerate during the charge/discharge process, and also ensures the long-term stability of WO_3_·2H_2_O/BC hybrids. All of these characteristics of WO_3_·2H_2_O/BC hybrids contribute to the high specific capacitance and good cycling stability.

## Conclusions

In summary, we successfully synthesized WO_3_·2H_2_O/BC hybrids via a facile γ-irradiation method. The electrochemical capacitive behaviors of the WO_3_·2H_2_O/BC hybrids and BC have been demonstrated in 6 M KOH electrolyte between −1 and 0 V. In comparison with the BC electrode, the WO_3_·2H_2_O/BC hybrid electrode showed a higher *C*_s_ (391 F g^−1^ at 0.5 A g^−1^) and superior cycling performance (approximately 82% retention even after 10,000 cycles). The excellent performance achieved by the WO_3_·2H_2_O/BC hybrid electrode is owing to the combined effect of BC with good conductivity and WO_3_·2H_2_O with superior pseudocapacitive behavior. This γ-irradiation method would also pave a new way of designing other conducting semiconductors as promising electrode materials with enhanced performance for energy storage device applications.

## Ethics statement

On behalf of, and having obtained permission from all the authors, I declare that: (a) the material has not been published in whole or in part elsewhere; (b) the paper is not currently being considered for publication elsewhere; (c) all authors have been personally and actively involved in substantive work leading to the report, and will hold themselves jointly and individually responsible for its content; I testify to the accuracy of the above on behalf of all the authors.

## Author contributions

YT developed the concept. XDL designed the experiments. FY and JJ conducted the experiments. RM and XCL built the cells and carried out the performance characterizations. ZF and CW supervised the research. FY and JJ co-wrote the manuscript. All authors discussed the results and commented on the manuscript.

### Conflict of interest statement

The authors declare that the research was conducted in the absence of any commercial or financial relationships that could be construed as a potential conflict of interest.
