# Supplementary material for: Fabrication of WO3·2H2O/BC Hybrids by the Radiation Method for Enhanced Performance Supercapacitors
Source: Front Chem. 2018 Aug 13;6:290. doi: 10.3389/fchem.2018.00290 (PMC6099569; doi:10.3389/fchem.2018.00290)
Supplement: Supplementary file 1 [file Presentation_1.PDF]

Supplementary Material

Fabrication of $WO_3 \cdot 2H_2O$/BC hybrids by radiation method for enhanced performance supercapacitors

Fan Yang[1†], Jinzhi Jia[1, 2†], Rui Mi[1], Xichuan Liu[1], Zhibing Fu[1], Chaoyang Wang[1], Xudong Liu[1,3]*, Yongjian Tang[1]*

*** Correspondence:** Xudong Liu: 8sliuxudong@caep.cn

Yongjian Tang: tangyongjian2000@sina.com

[†] **These authors contributed equally to this work.**

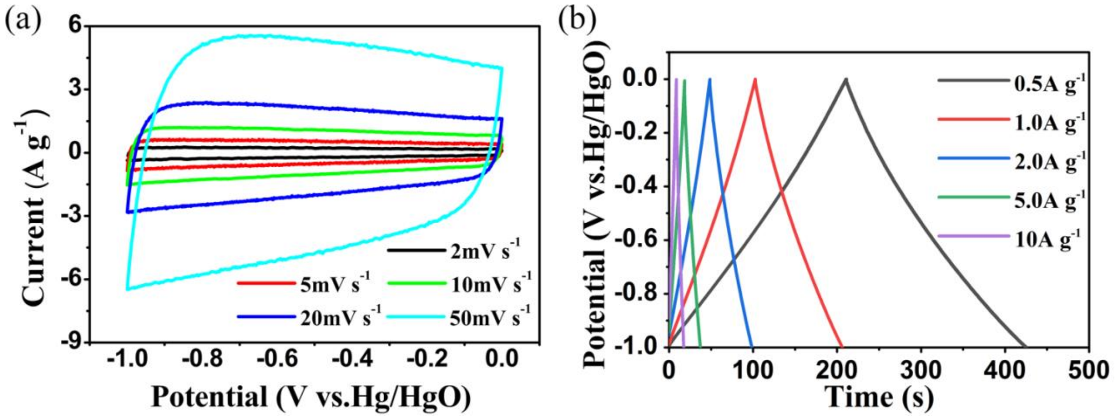

FIGURE S1 (a) The CV curves of the BC electrode at different scan rates, and (b) GCD curves of the BC electrode at different current densities.

Table S1 Comparison of various WO_3-carbon based electrodes in recent years as supercapacitors.

Sr. No.	Material	Synthesis method	Specific capacitance	Cycling Stability	Ref.
1	WO_3 nanowires arrays/carbon cloth	Hydrothermal method	521 F g^{-1} at 1 A g^{-1}	2000 (100%)	(Liu et al., 2017)
2	WO_3 in carbon aergel	Thermal method	609 F g^{-1} at 5 mA s^{-1}	1000 (98%)	(Liu et al., 2018)
3	WO_3 in carbon aergel	Thermal method	700F g^{-1} at 25 mA s^{-1}	4000 (95%)	(Wang et al., 2014)
4	Graphene nanosheets-WO_3	Thermal method	143.6 F g^{-1} at 0.1 A g^{-1}	—	(Cai et al., 2014)
5	WO_3 coated graphene nanosheet	Electrostatic method	495 F g^{-1} at 1 A g^{-1}	1000 (87.5%)	(Qiu et al., 2016)
6	Graphene - WO_3	Hydrothermal method	465 F g^{-1} at 1 A g^{-1}	2000 (97.7%)	(Nayak et al., 2017)
7	Gr- WO_3 hybrids	Hydrothermal method	580 F g^{-1}	1000 (92%)	(Xing et al., 2016)
8	$WO_3{\cdot}2H_2O$/BC	γ-irradiation method	391 F g^{-1} at 0.5 A g^{-1}	10,000 (82%)	This work

Cai, Y., Wang, Y., Deng, S., Chen, G., Li, Q., Han, B., Han, R., Wang, Y.,et al. (2014). Graphene nanosheets-tungsten oxides composite for supercapacitor electrode. *Ceram. Int.* 40, 4109-4116. doi: 10.1016/j.ceramint.2013.08.065

Liu, B., Wang, Y., Jiang, H., Zou, B. X., (2017). WO_3 Nanowires on Graphene Sheets as Negative Electrode for Supercapacitors. *J. Nanomater*. 24, 109-117. doi: 10.1155/2017/2494109

Liu, X. D., Sheng, G., Zhong, M. L., and Zhou, X. W., (2018). Hybrid nanowires and nanoparticles of WO_3 in a carbon aerogel for supercapacitor applications. *Nanoscal* 10, 4209-4217. doi: 10.1039/c7nr07191d

Nayak, A. K., Das, A. K., and Pradhan, D. (2017). High Performance Solid-State Asymmetric Supercapacitor using Green Synthesized Graphene-WO_3 Nanowires Nanocomposite. *Acs Sustainable Chem. Eng.* 5, 10128-10138. doi: 10.1021/acssuschemeng.7b02135

Qiu, M., Sun, P., Shen, L., Wang, K., Song, S., Yu, X., et al. (2016). WO_3 nanoflowers with excellent pseudo-capacitive performance and the capacitance contribution analysis. *J. Mater. Chem.* 4, 7266-7273. doi: 10.1039/c6ta00237d

Wang, Y., Wang, C., Cheng, W., Lu, S., et al. (2014). Dispersing WO_3 in carbon aerogel makes an outstanding supercapacitor electrode material. *Carbon* 69, 287-293. doi: 10.1016/j.carbon.2013.12.027

Xing, L.L., Huang, K.J., and Fang, L.X., et al. (2016). Preparation of layered graphene and tungsten oxide hybrids for enhanced performance supercapacitors. *Dalton Trans.* 45, 17439-17446. doi: 10.1039/c6dt03719d
